# Supplementary material for: Enhancer hijacking activates oncogenic transcription factor NR4A3 in acinic cell carcinomas of the salivary glands
Source: Nat Commun. 2019 Jan 21;10:368. doi: 10.1038/s41467-018-08069-x (PMC6341107; doi:10.1038/s41467-018-08069-x)
Supplement: Supplementary file 1 — Supplementary Information [file 41467_2018_8069_MOESM1_ESM.pdf]

Supplementary Figure 1. High resolution image of NR4A3 break apart FISH demonstrating split signals indicating a genomic rearrangement in the majority of the cells.

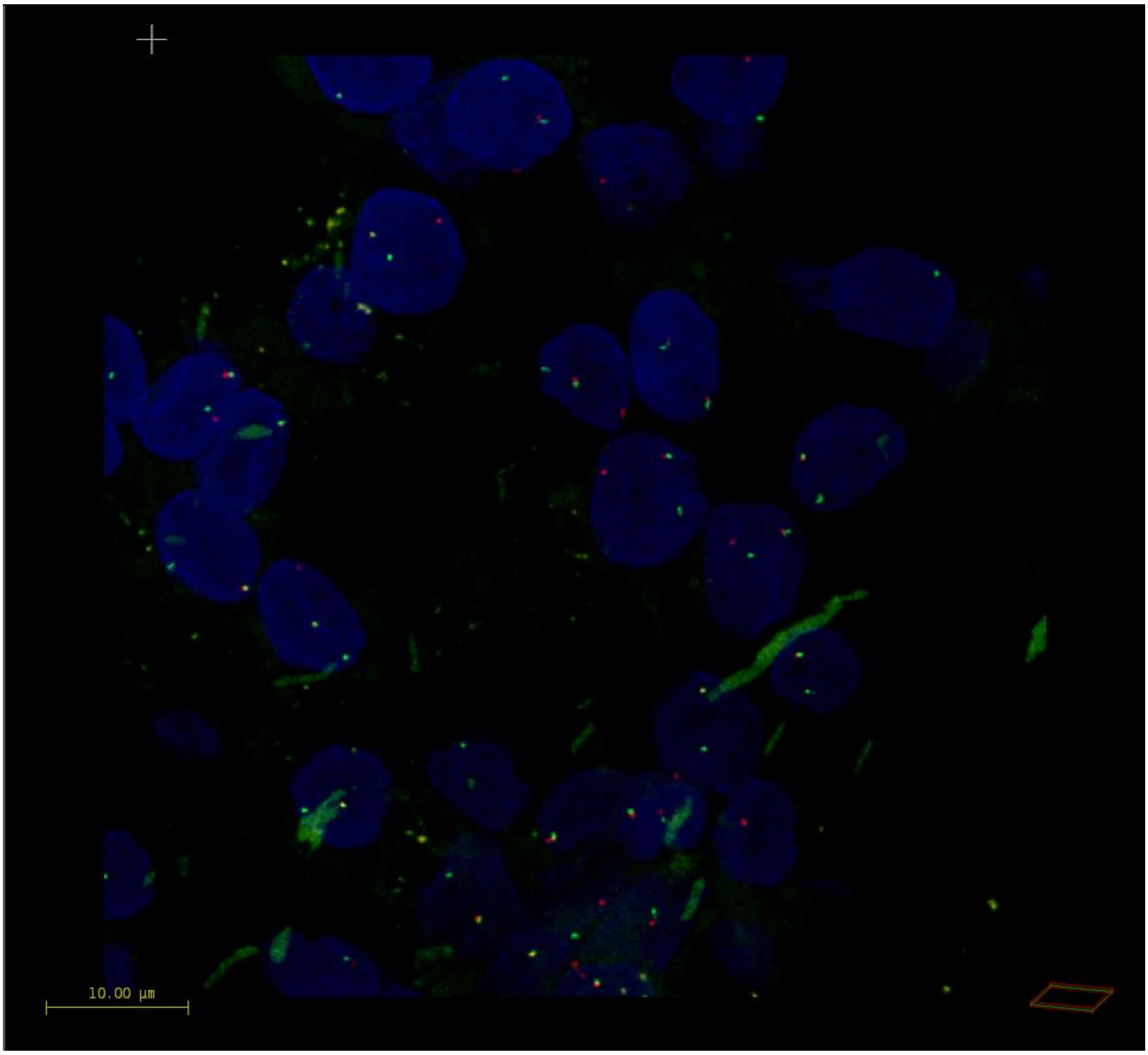

The image was obtained with Leica SP5 II, Software Revision 2.6.3.1873 using a glycerol objective lens (63x, NA 1.3), detection with PMTs. Nyquist criterion matched for xy and z for all three channels, super critical pinhole with 0.8 Airy Units each channel. Stacks were post processed using Huygens Prof. Vers. 18.04 with deconvolution parameters: CMLE algorithm, SNR:20, 40 iterations.

Supplementary Figure 2. Hockey stick plots of active H3K27ac chromatin marks in three AciCC tumor samples

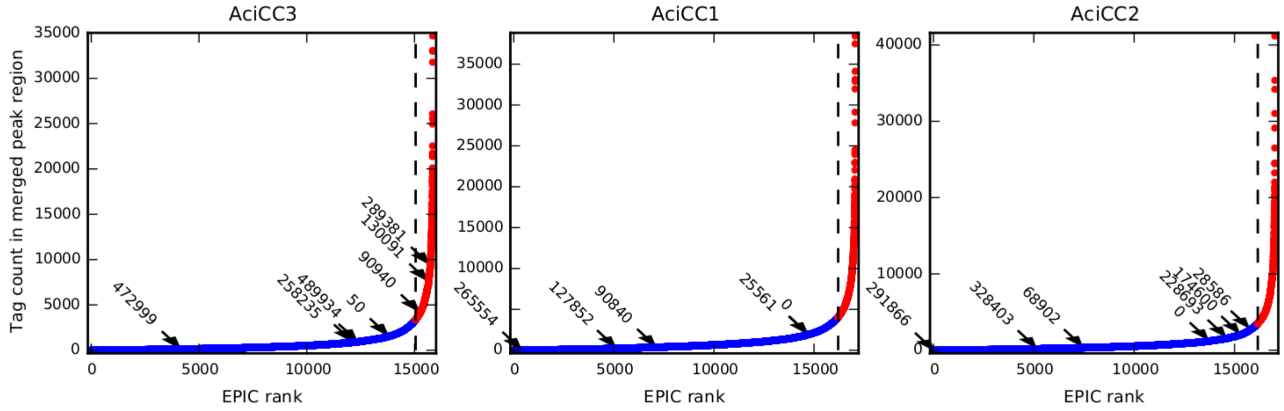

Putative enhancer regions from the 4q13 SCPP gene cluster are indicated as basepair counts upstream of the translocation break point. Potential super-enhancers defined as the 5% strongest chromatin marks are indicated in red.

Supplementary Figure 3. Comparison of differentially expressed genes and ChIP-seq peaks for H3K27ac and NR4A3.

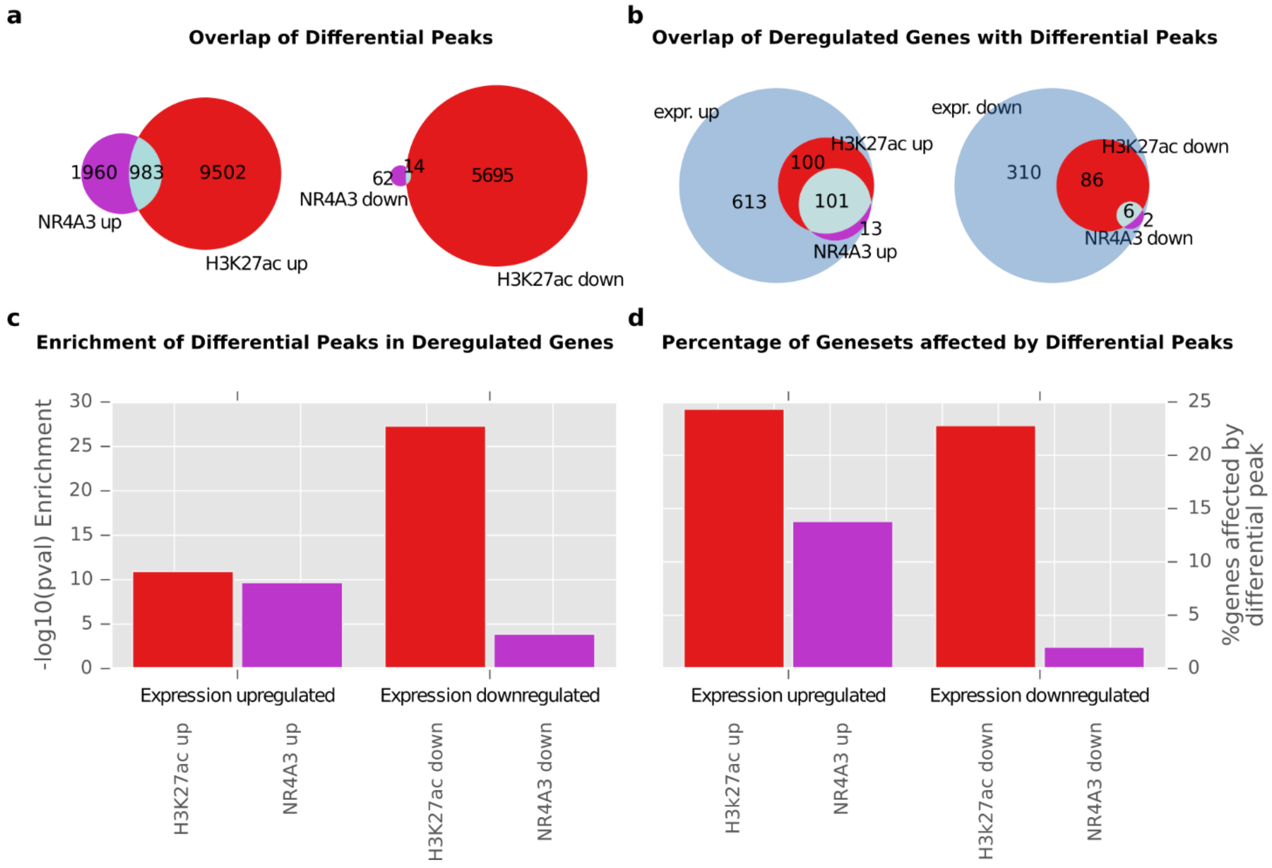

a, Venn diagram showing overlap of upregulated (left) or downregulated (right) H3K27ac and NR4A3 peaks. b, Venn diagram showing overlap of upregulated (left) or downregulated (right) genes associated with upregulated or downregulated H3K27ac and NR4A3 peaks. c, Enrichment of H3K27ac and NR4A3 upregulated peaks among upregulated genes (left), and of H3K27ac and NR4A3 downregulated peaks among downregulated genes (right). d, Percentage of upregulated (left) and downregulated (right) genes with corresponding change of H3K27ac and NR4A3 peaks.

Supplementary Figure 4. Boxplots and raw values of mRNA expression (FPKM) for NR4A3, CCND1 and ENO3 in ten AciCCs vs. three normal parotid gland samples.

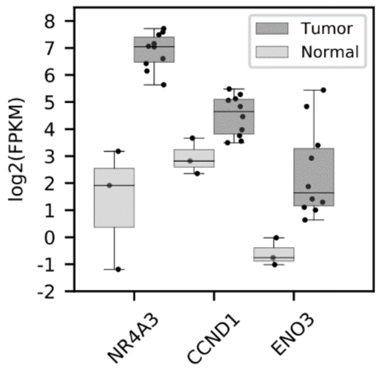

Box-plot centre line: median; bounds of box: 25 and 75 % quantiles; whiskers: minimum and maximum values). deSeq2 Test for differential expression; NR4A3 P adjusted=2.992e-07 , CCND1 P adjusted=0.15, ENO3 P adjusted=0.009. Source data are provided as a Source Data file.

Supplementary Figure 5. Representative immunohistochemical images of NR4A3, CCND1 and ENO3 in AciCC and normal parotid gland tissues.

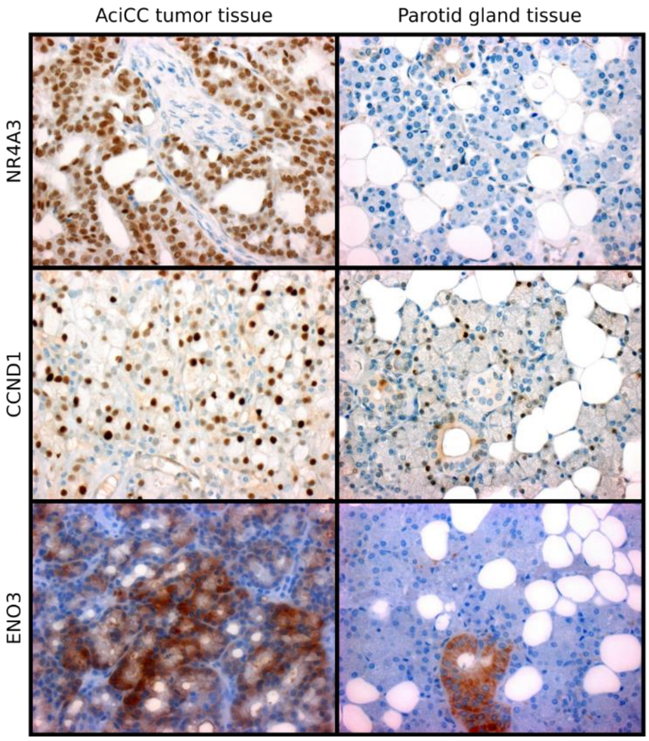

Supplementary Figure 6. Scatter plot of FPKM values for RNA-seq data from immortalized mouse salivary gland cells

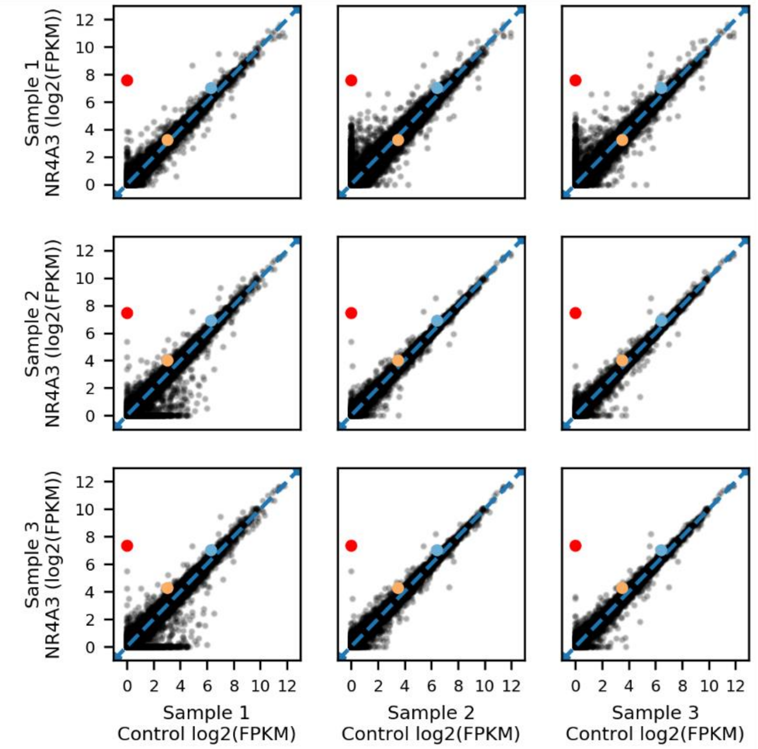

Shown are scatter plots of FPKM values for RNA-seq data from immortalized mouse salivary gland cells from three replicates stably transduced with NR4A3 vs. three replicates stably transduced with red firefly luciferase (control). Human NR4A3 is marked in red, and mouse CCND1 and ENO3 are marked in blue and orange, respectively. Source data are provided as a Source Data file.

Supplementary Figure 7. Scatter plot of LFQ-intensities values for proteins detected by mass spectrometry

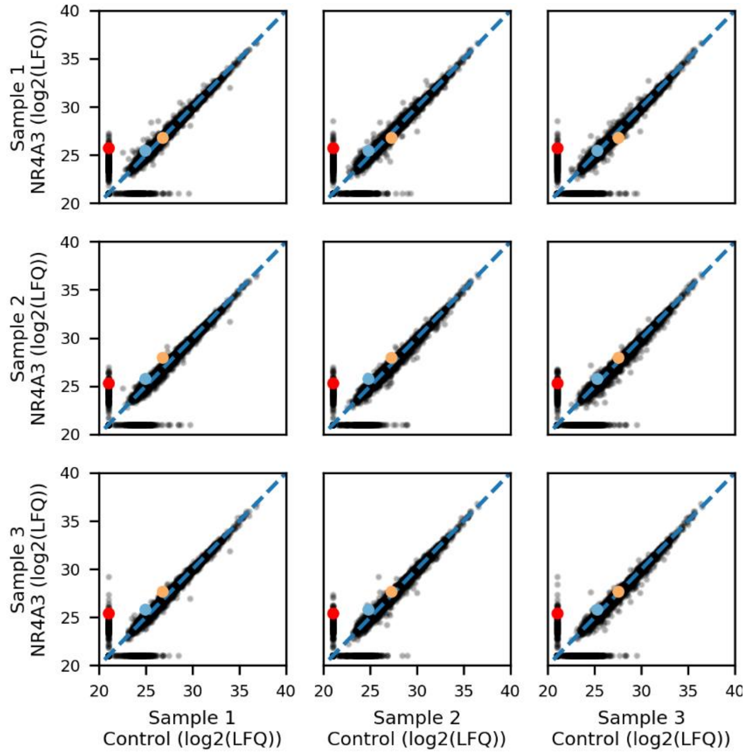

Shown are scatter plots of LFQ-intensities values for proteins detected by mass spectrometry from immortalized mouse salivary gland cells comparing three replicates stably transduced with NR4A3 vs. three replicates stably transduced with red firefly luciferase (control). Human NR4A3 is marked in red, and mouse CCND1 and ENO3 are marked in blue and orange, respectively. Source data are provided as a Source Data file.

Supplementary Figure 8. qRT-PCR data for NR4A3 target genes Ccnd1 and Eno3 from immortalized mouse salivary gland cells

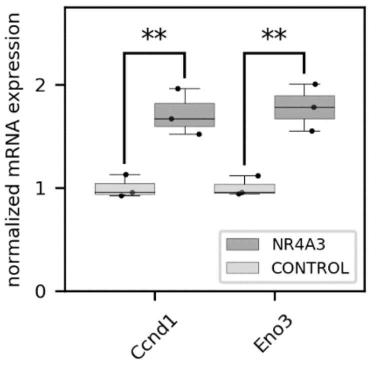

Shown are three replicates stably transduced with NR4A3 vs. three replicates stably transduced with red firefly luciferase (control). Box-plot centre line: median; bounds of box: 25 and 75 % quantiles; whiskers: minimum and maximum values, Two-tailed Student's t-test; **: $P<0.01$. Source data are provided as a Source Data file.

Supplementary Figure 9. CCND1 Western blot analysis

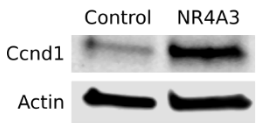

CCND1 Western blot analysis comparing immortalized mouse salivary gland cells stably transduced with NR4A3 or red firefly luciferase (control) open reading frames. The original blot images are included in the Source Data file.

Supplementary Figure 10. qPCR validation of NR4A3 overexpression in MCF10A cells.

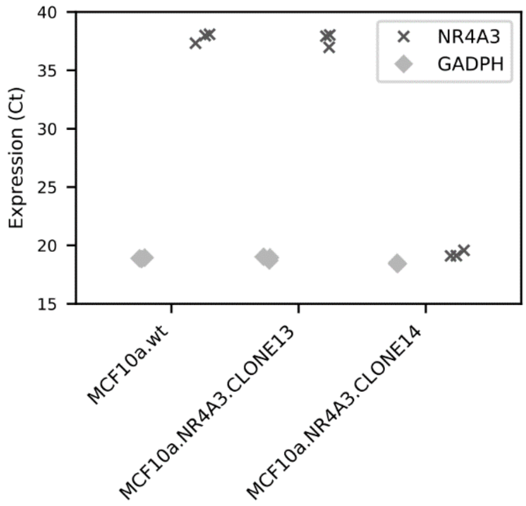

Shown are the raw CT values for NR4A3 and GAPDH in MCF10A wildtype cells and MCF10A cells transduced with NR4A3 open reading frames. Clone 13 displayed no NR4A3 expression and was used as negative control, whereas Clone 14 showed high NR4A3 expression levels and was used as NR4A3 positive control.

Supplementary Figure 11. Representative example of gating strategy.

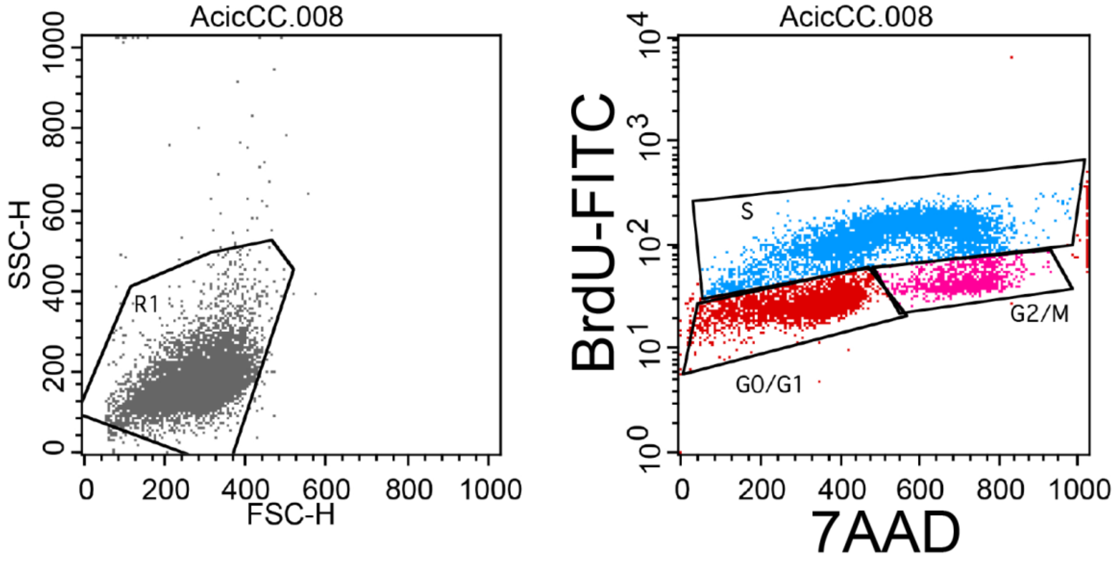

Main cell population was gated in FSC/SSC excluding debris and douplets. Cells were defined to be in G1-phase when they had low signals in both channels, G2-phase when they were high in 7-AAD but low in BrdU-FITC, and S-phase when they had positive signals in BrdU-FITC.

Supplementary Table 1. List of exonic variants in four AciCCs with paired tumor-normal whole genome sequencing data.

ID	CHR.	POS	REF>ALT	GENE	cDNA	Protein
1	1	23688964	C>T	ZNF436	c.G911A	p.R304K
1	5	16465192	C>T	ZNF622	c.G583A	p.E195K
1	5	94957126	G>A	GPR150	c.G1147A	p.D383N
1	6	30918925	T>C	DPCR1	c.T2684C	p.L895P
1	11	78433711	A>G	TENM4	c.3800+2T>C	splice site
1	12	53343124	G>A	KRT18	c.G167A	p.G56D
1	11	64083295	CGGG>C	ESRRA	c.1127_1129del	p.376_377del
1	16	66974138	TC>T	CES2	c.630delC	p.I210fs
2	1	169510367	T>G	F5	c.A3976C	p.I1326L
2	2	54021528	C>A	ERLEC1	c.C209A	p.T70K
2	2	73677676	G>C	ALMS1	c.G3893C	p.G1298A
2	2	74425868	C>T	MTHFD2	c.C100T	p.R34X
2	2	133175291	G>A	GPR39	c.G676A	p.G226S
2	2	220354216	A>C	SPEG	c.A8476C	p.T2826P
2	2	238285621	C>T	COL6A3	c.G1643A	p.R548H
2	4	100572808	T>C	RP11-766F14.2	c.A2998G	p.K1000E
2	5	37016264	A>A	NIPBL	c.A4768G	p.R1590G
2	5	129037090	G>A	ADAMTS19	c.G2946A	p.M982I
2	6	131222297	C>T	EPB41L2	c.G953A	p.R318Q

2	6	159188007	G>A	EZR	c.C1700T	p.T567M
2	8	10756078	C>T	XKR6	c.G1310A	p.R437Q
2	8	56436630	G>C	XKR4	c.G1797C	p.L599F
2	11	73431893	T>A	RAB6A	c.A181T	p.T61S
2	14	65252254	C>T	SPTB	c.3855+1G>A	splice site
2	X	111090589	A>C	TRPC5	c.T1453G	p.S485A
2	5	668526	C>CT	AC026740.1	c.432_433insT	p.T144fs
2	6	33237584	G>GGCGACCACC	VPS52	c.239_240insGGTGGTCGC	p.L80delinsLVVA
2	8	37623073	GGC>G	PROSC	c.130_131del	p.A44fs
2	8	104427572	TG>T	DCAF13	c.355delG	p.G119fs
2	9	20414343	ACTG>A	MLLT3	c.498_500del	p.166_167del
2	14	21883889	AGAACTGCATGGAAGGCAAAGTCTCGCC>A	CHD8	c.1867_1893del	p.623_631del
2	16	69143493	GGAGC>G	HAS3	c.196_199del	p.E66fs
3	1	44466837	T>C	SLC6A9	c.A1391G	p.Q464R
3	2	63283273	A>G	OTX1	c.A887G	p.H296R
3	2	130832598	C>T	POTEF	c.G2447A	p.R816H
3	3	194790511	T>C	XXYLT1	c.A506G	p.Y169C
3	4	8608534	C>T	CPZ	c.C977T	p.P326L
3	7	99700527	G>T	AP4M1	c.G295T	p.E99X
3	9	139906197	T>C	ABCA2	c.A5627G	p.N1876S
3	11	828704	C>T	EFCAB4A	c.C97T	p.Q33X
3	11	1908050	C>T	LSP1	c.C620T	p.T207M
3	12	53470954	C>G	SPRYD3	c.G115C	p.A39P

3	12	80729863	C>T	OTOGL	c.C4516T	p.R1506X
3	13	38211256	A>T	TRPC4	c.T2733A	p.C911X
3	17	813084	G>T	RP11-676J12.7	c.G36T	p.M12I
3	19	12807203	G>A	FBXW9	c.C193T	p.P65S
3	19	15636205	C>T	CYP4F22	c.C58T	p.R20C
3	X	53571589	T>C	HUWE1	c.A11183G	p.Q3728R
3	X	102317949	A>G	BEX1	c.T254C	p.I85T
3	2	230923806	ATACCCACTATCAAG>A	SLC16A14	c.249_259del	p.T83fs
3	17	39432220	TGTG>T	KRTAP9-7	c.272_274del	p.91_92del
4	1	51767929	A>C	TTC39A	c.T1087G	p.W363G
4	2	135712060	G>A	CCNT2	c.G2035A	p.G679R
4	2	179247325	A>C	OSBPL6	c.A1511C	p.H504P
4	2	179404879	T>A	TTN	c.A70819T	p.R23607W
4	3	49700819	C>G	BSN	c.C11228G	p.A3743G
4	5	66461698	T>G	MAST4	c.T6700G	p.S2234A
4	5	132101112	A>G	SEPT8	c.T140C	p.I47T
4	5	140516055	A>C	PCDHB5	c.A1039C	p.T347P
4	16	58060288	G>A	MMP15	c.G34A	p.G12S
4	16	70432214	G>A	ST3GAL2	c.C220T	p.R74C
4	19	831737	G>T	AZU1	c.G616T	p.V206F
4	21	30698468	A>G	BACH1	c.A323G	p.K108R
4	X	10096670	G>A	WWC3	c.G2354A	p.R785Q
4	6	157100023	CGGA>C	ARID1B	c.961_963del	p.321_321del

4	7	154760608	TCTG>T	PAXIP1	c.1300_1302del	p.434_434del

Supplementary Table 2. Summary of clinico-pathological data and immunohistochemical findings in 29 patients with AciCC.

ID	S/A	Loc/Size	pT,pN,R	Tissue/Grad	NR4A3 FISH	NR4A3	CCND1 (%)	ENO3	Follow-up (mo)	Status (mo)
1	f/34	p/0.7	pT1,pNx,R0	prim./low	break	2+	50	0	no events	NED (5)
2	m/31	p/1.2	pT1,pN0,R0	prim./low	break	2+	80	3+	no events	NED (41)
3	f/32	p/1.5	pT1,pN0,R0	prim./low	break	3+	80	0	no events	NED (2)
4	f/60	p/1.5	pT1,pN0,R0	prim./low	normal	2+	80	3+	no events	NED (11)
5	f/49	p/1.7	pT1,pNx,Rx	prim./low	break	3+	40	0	no events	NED (1)
6	m/59	p/1.7	pT1,pN1,R0	prim./low	break	3+	80	2+	no events	NED (44)
7	f/55	p/1.8	pT1,pN0,R0	prim./low	single red	3+	40	1+	no events	NED (7)
8	f/22	p/1.8	pT1,pNx,R0	prim./low	break	2+	50	0	no events	NED (33)
9	m/68	p/2.5	pT2,pN1,R0	prim./low	NA	1+	90	1+	no events	NED (24)
10	m/59	p/2.6	pT2,pN1,R0	prim./low	break	2+	50	1+	no events	NED (6)
11	m/20	p/2.8	pT2,pN0,R0	prim./low	break	3+	60	1+	no events	NED (29)
12	f/43	p/2.8	pT2,pN0,R0	prim./low	break	2+	70	1+	no events	NED (5)
13	f/42	p/3.0	pT2,pN0,R0	prim./low	break	2+	60	1+	no events	NED (11)
14	f/91	p/3.2	pT3,pN0,R0	prim./low	break	3+	70	2+	no events	DOOC (18)
15	m/65	p/3.4	pT3,pN1,R0	prim./low	normal	2+	40	2+	no events	NED (9)
16	f/68	p/3.5	pT4a,pN0R0	prim./low	break	3+	20	1+	no	NED

									events	(23)
17	m/77	p/3.5	pT2,pN0/R0	prim./low	break	2+	50	2+	no events	DOOC (47)
18	f/72	p/6.0	pT3,pN0,R0	prim./low	break	3+	50	2+	no events	NED (27)
19	f/55	p/1.3	pN1,pN0,R0	prim./low	break	2+	80	1+	rec (129)	AWD (143)
20	m/56	p/4.5	pT3,pN0,R0	prim./low	break	3+	50	2+	rec (28)	AWD (92)
21	f/26	p/4.5	pT3,pN0,R0	prim./low	break	2+	40	3+	rec (3), met (14)	AWD (60)
22	m/45	na	pTx,pNx,Rx	rec./low	normal	0	70	1+	rec (120)	AWD (148)
23	m/78	na	pTx,pNx,Rx	rec./low	break	2+	70	1+	rec (48)	AWD (94)
24	f/45	na	pTx,pNx,Rx	rec./high	normal	1+	80	1+	rec (23)	AWD (108)
25	f/48	p/1.5	pT1,pN2b,R0	prim./high	single red	3+	60	1+	met (9)	DOT (18)
26	f/74	p/4.5	pT3,pN2b,R0	prim./high	single red	3+	60	3+	met (37)	AWD (36)
27	f/70	p/5.8	pT3,pN2b,R1	prim./high	break	3+	60	1+	met (0)	NA (0)
28	f/78	p/3.9	pT2,pN2b,R0	prim./high	break	2+	60	0+	no events	NED (11)
29	f/68	p/2.5	pT2,pNx,R1	prim./high	break	3+	40	2+	rec (5)	DOT (10)

s, sex; a, age; loc, location; Grad, Grading; mo, months; f, female; m, male; p, parotis; prim, primary; rec, recurrence; NED, no evidence of disease; DOOC, died of other cause; AWD, alive with disease; DOT, died of tumor.

Supplementary Table 3. List of proteins exclusively detected by mass spectrometry (LFQ-intensities) either in mouse primary submandibular gland cells upon stable overexpression of human NR4A3 or red firefly luciferase control.

<u>Proteins expressed in NR4A3 overexpressing cells only</u>

NR4A3

Aldh3a1

Lsm8

Snx30

Mfge8

Prkag2

Abi2

Trpv4

Zfp106;Znf106

<u>Proteins expressed in control cells only</u>

Optn

Wbp2

Ralgps2

Mcts2

Chmp5

Cask

Pstpip2

Sod2

Fbxo3

Gid8

Vav2

Rrs1

Bag2

Mrps9

Mak16

Pdxp

Cggbp1
